# Mining Seasonal Marine Microbial Pattern with Greedy Heuristic Clustering and Symmetrical Nonnegative Matrix Factorization

**DOI:** 10.1155/2014/189590

**Published:** 2014-04-27

**Authors:** Fei Liu, Shao-Wu Zhang, Ze-Gang Wei, Wei Chen, Chen Zhou

**Affiliations:** ^1^College of Automation, Northwestern Polytechnical University, Xi'an 710072, China; ^2^Key Laboratory of Information Fusion Technology, Ministry of Education, Xi'an 710072, China

## Abstract

With the development of high-throughput and low-cost sequencing technology, a large number of marine microbial sequences were generated. The association patterns between marine microbial species and environment factors are hidden in these large amount sequences. Mining these association patterns is beneficial to exploit the marine resources. However, very few marine microbial association patterns are well investigated in this field. The present study reports the development of a novel method called HC-sNMF to detect the marine microbial association patterns. The results show that the four seasonal marine microbial association networks have characters of complex networks, the same environmental factor influences different species in the four seasons, and the correlative relationships are stronger between OTUs (taxa) than with environmental factors in the four seasons detecting community.

## 1. Introduction

The oceans cover approximately 139 million square miles—roughly 71% of the earth's surface. Marine microbes are the important composition in the marine ecosystem. They can provide the basis for the ocean's food webs and facilitate the flow of nitrogen, carbon, and energy in the ocean. Yet specific ecological relationships among these taxa and environment factors are largely unknown. This is partly due to the dilute, microscopic nature of the planktonic microbial community, which prevents direct observation of their interactions [1]. Although the technologies of microbial cultivation, gene chip, and metagenomics [2–4] can provide the information on microorganisms' potential ecological roles, they cannot describe the interactions among microbes and environment.

With the development of high-throughput DNA sequencing technologies that yield a mass of reads of rRNA (16S rRNA/18S rRNA) and DNA, we can describe the compositions of microbial communities, their diversity, and how communities change across space, time, or experimental treatments based on these sequence data [5]. However, most of the current analytical approaches often focus on the total numbers of taxa, the relative abundances of individual taxa, and the extent of phylogenetic or taxonomic overlap between communities or community categories [6–8]. In contrast, there has been far less attention focused on using sequence data to explore the direct or indirect relationship among microbial taxa and environments. Some researchers used the network analysis to explore cooccurrence pattern in soil and ocean [9–11], but they just constructed the association networks to show the cooccurrence pattern and did not further mine the networks to find the pattern structures. The microbial association (or cooccurrence) patterns can offer new insight into the structure of complex microbial communities, revealing the niche spaces shared by community members and identifying habitat affinities or shared physiologies that could guide more experimental settings.

In this paper, we proposed a novel method called HC-sNMF to detect the association community patterns and structures in the four seasonal marine networks. HC-sNMF provides new insights into the natural history of microbes, finding the relationship among microbes and environmental factors and trying to determine the microbial association pattern difference among seasons and which environmental factors might have the greatest influence on the varying diversity.

## 2. Material and Methods

### 2.1. Dataset

The 16S rRNA sequence dataset used in this paper was downloaded from http://vamps.mbl.edu/index.php, which includes 969,400 sequences generated from 76 time point seawater samples at the surface of L4 sampling site in the West English Channel [10]. The 76 seawater samples were arranged into winter (January–March), spring (April–June), summer (July–September), and fall (October–December) seasons, in which 16, 24, 21, and 15 samples belong to winter, spring, summer, and fall seasons, respectively. And the 16S rRNA sequence numbers of winter, spring, summer, and fall seasons are 231,640, 276,932, 247,907, and 212,921, respectively. In order to establish the seasonal association networks of microbe and environmental factor at the taxonomic level (e.g., species, genus), the 16S rRNA sequences were grouped into species-level operational taxonomic units (OTUs) with NbHClust algorithm, which resulted in 6,793 OTUs.

### 2.2. HC-sNMF Work Engine and Process

**Figure 1:**
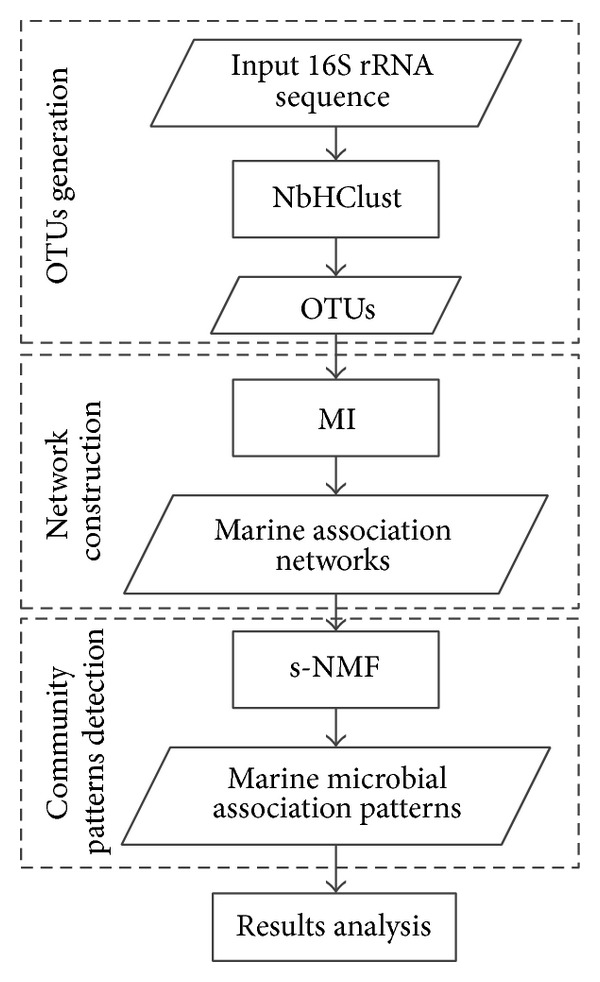
The flowchart showing the work process of HC-sNMF.

The work engine and process of HC-sNMF consist of the three following parts: (i) OTUs generation with NbHClust algorithm, (ii) network construction with mutual information algorithm, and (iii) community patterns detection with symmetrical nonnegative matrix factorization method.  Figure 1 is a flowchart showing the work process of the HC-sNMF.

### 2.3. NbHClust Algorithm

For OTU inflation caused by 454 sequencing errors, we proposed a heuristic clustering method based on neighbor seeds, namely, NbHCluster. Based on the distribution of homopolymer, the idea of neighbor sequence was introduced to generated neighbor seeds. Then, a heuristic cluster strategy was used to cluster the sequences based on neighbor seeds instead of single seed. Finally, a constraint parameter based on cluster size was used to fine the clusters. The pseudocode of NbHClust is as shown in Pseudocode 1.

**Figure:**
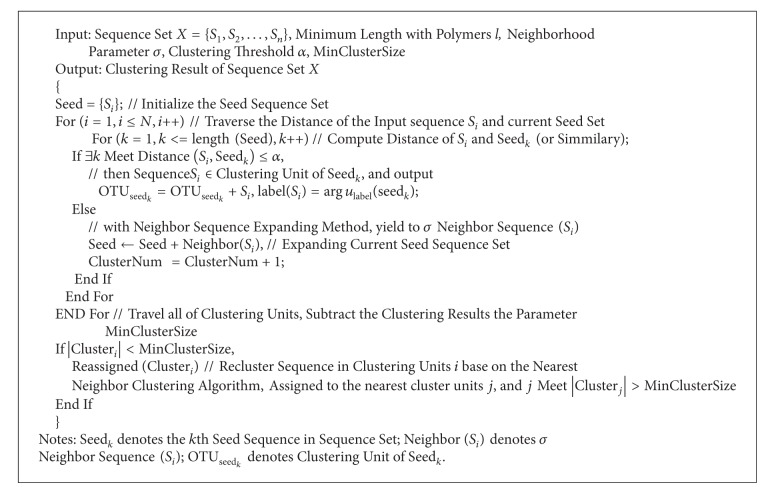
Pseudocode 1

### 2.4. Networks Construction

In order to research the association among different microbial species and environmental factors, we use vectors *X*_*μ*_ and *X*_*ν*_ to represent OTU and environmental factor in the four seasons, respectively,

(1) Xμ=[xμ1,xμ2,…,xμs,…,xμS], (μ=1,…,6739),Xν=[xν1,xν2,…,xνs,…,xνS], (ν=1,…,18),

where *x*_*μs*_ is the *μ*th OTU abundance value in the* s*th sampling; that is, *x*_*μs*_ equals the ratio of the sequence number *N*_*μs*_ contained in the *μ*th OTU and the total sequence number *N*_*s*_ contained in the* s*th sampling. To reduce the sequencing effort bias, the *x*_*μs*_ value was set to zero if *N*_*μs*_ < 5. For reducing the false higher correlation between vectors, we also remove these OTU vectors which contain less than 3 nonzero elements. After this processing, we can obtain 1,212 OTU vectors, in which spring season contains 280, summer 254, fall 313, and winter 365 OTUs, respectively. *x*_*νs*_ is the environmental factor variable such as serial day (E1), day length (E2), DX1 (E3), DX2 (E4), photosynthetically active radiation (E5), North Atlantic Oscillation data (E6), primary productivity (E7), daily primary productivity (E8), mixed layer depth (E9), the concentrations of ammonia (E10), chlorophyll (E11), NO_2_ + NO_3_ (E12), salinity (E13), silicate (E14), SRP (E15), temperature (E16), total organic carbon (E17), and total organic nitrogen (E18) [10]. Then, the four microbial abundance matrixes and four environment factor matrixes of spring, autumn, fall, and winter seasons were constructed by normalizing every OTU and environment factor vector with zero-mean normalization method.

Beyond Pearson correlation, mutual information (MI) can capture nonlinear dependencies and topology sparseness between variables. Here, we used MI [11] to compute the association relationship between variables and construct the seasonal marine microbial association networks. The process of MI can be described simply as follows.

Suppose that *D* is the value range of variable *X* and the subinterval set {*D*_*λ*_}, *λ* = 1,2,…, *M*, is a partition of *D*, satisfying that ∪_*λ*_{*D*_*λ*_} = *D* and *D*_*λ*_∩*D*_*γ*_ = *ϕ*  if  *λ* ≠ *γ*. Define the following two delta functions:

(2) δ(xs,Dλ)={1,if  xs∈Dλ0,else,δ(xs,ys,Dλ,Dγ)={1,if  xs∈Dλ,  ys∈Dγ0,else(s=1,2,…,S; λ,γ=1,2,…,M).

The probability of {*D*_*λ*_} according the variable *X* and the joint probability of {*D*_*λ*_, *D*_*γ*_} according to variables *X* and *Y* are defined as

(3) $$\begin{array}{c} p_{X}\left(D_{\lambda }\right)=\frac{1}{S}\overset{S}{\underset{s=1}{\sum }}\delta \left(x_{s},D_{\lambda }\right),\\ p_{X,Y}\left(D_{\lambda },D_{\gamma }\right)=\frac{1}{S}\overset{S}{\underset{s=1}{\sum }}\delta \left(x_{s},y_{s},D_{\lambda },D_{\gamma }\right). \end{array}$$

The entropy and joint entropy of *X* and *Y* are defined as

(4) $$\begin{array}{c} H\left(X\right)=-\overset{M}{\underset{\lambda =1}{\sum }}p_{X}\left(D_{\lambda }\right)\log p_{X}\left(D_{\lambda }\right),\\ H\left(Y\right)=-\overset{M}{\underset{\lambda =1}{\sum }}p_{Y}\left(D_{\lambda }\right)\log p_{Y}\left(D_{\lambda }\right),\\ H\left(X,Y\right)=-\overset{M}{\underset{\lambda =1}{\sum }}\;\overset{M}{\underset{\gamma =1}{\sum }}p_{X,Y}\left(D_{\lambda },D_{\gamma }\right)\log{p_{X,}}_{Y}\left(D_{\lambda },D_{\gamma }\right). \end{array}$$

So, we can calculate the mutual information between two variables *X* and *Y* according to the following formulate:

(5) $$\begin{array}{c} I\left(X,Y\right)=H\left(X\right)+H\left(Y\right)-H\left(X,Y\right). \end{array}$$

The permutation test was used to calculate the statistical significance. We considered that there are robust associations between OTU-OTU and OTU environmental factor vector if *P* value ≤ 0.01, and there is a robust association between environmental factor vectors if *P* value ≤ 0.05. In the end, we can construct the four marine microbial association networks (Figure 4) of spring, summer, fall, and winter seasons. These networks are weighted and undirected networks in which the edge weight is MI value of two variables (nodes).

### 2.5. Symmetrical Nonnegative Matrix Factorization (s-NMF) Clustering Algorithm

For a weighted and undirected graph *G*(*V*, *E*) with *n* nodes and *l* links, we can describe it by a weighted adjacency matrix *A* = [*A*_*ij*_]_*n*×*n*_, where *A*_*ij*_ ≥ 0. Let *O* be the feature matrix of graph *G* calculated from *A*, and *O* represents the node-node similarity.

Suppose that *n* nodes can be grouped into *r* overlapping cliques (or communities). Then, a clique-node similarity matrix *W* = [*W*_*ki*_]_*r*×*n*_ was introduced to represent the similarity degree between node and clique. *W*_*ki*_ indicates the closeness degree between node *i* and clique *k*. Here, *W* is nonnegative matrix, reflecting the relationship between node and clique. Because ∑_*k*=1_^*r*^ *W*_*ki*_ *W*_*kj*_ is an approximation of similarity between node *i* and node *j*, and *Z* also represents the node-node similarity; thus, we can use *Z*_*ij*_ to estimate ∑_*k*=1_^*r*^ *W*_*ki*_ *W*_*kj*_. Our task can now be summarized as computing the parameter *W* so as to minimize the function *F*_*G*_:

(6) min⁡W≥0 FG(O,W)=||O−WTW||F2=12∑ij[(O−WTW)∘(O−WTW)]ij,

where *A*∘*B* is the Hadamard product (or element-by-element product) of matrices *A* and *B*. To solve this optimization problem, we will introduce a symmetrical nonnegative matrix factorization (*s*-NMF) method which is an improved method of nonnegative matrix factorization [12]. NMF can be described as a linear decomposition *O* ≈ *W* ^*T*^ *H*, where *O* ∈ *R* ^*n*×*m*^ is a positive matrix and *W* ∈ *R* ^*r*×*n*^ and *H* ∈ *R* ^*r*×*m*^ are nonnegative matrices. *W* and *H* are iteratively updated according to the following rules [13, 14]:

(7) $$\begin{array}{c} H_{k+1}=H_{k}\circ \frac{\left[W_{k}O\right]}{\left[W_{k}W_{k}^{T}H_{k}\right]}, \end{array}$$

(8) $$\begin{array}{c} W_{k+1}=W_{k}\circ \frac{\left[H_{k}O\right]}{\left[H_{k}H_{k}^{T}W_{k}\right]}, \end{array}$$

where [*A*]/[*B*] is the Hadamard division (or element-by-element division) of matrices *A* and *B*.

Supposing that *H* = *W*,* s*-NMF can be seen as a constraint form of NMF. Thus, the iteratively updated rule of* s*-NMF can be described as follows:

(9) $$\begin{array}{c} W_{k+1}=W_{k}\circ \frac{\left[W_{k}O\right]}{\left[W_{k}W_{k}^{T}W_{k}\right]}. \end{array}$$

Obviously, the optimal solution of* s*-NMF is a subset of the NMF solution set. The stable points of (8) can only fall into the set of NMF's stationary points which satisfy *H* = *W*, hence guaranteeing the convergence of* s*-NMF.

By normalizing the column of *W*, we can obtain the fuzzy membership degree matrix *U*. Then, the clique corresponding to the largest element of each column in *U* is determined as the final membership clique of each node. That is, if *U*_*ki*_ is the maximum in the column* i*, the node *i* is classified as the clique *k*.

In order to determine the optimal number of community *r*, we iteratively increase *r* and choose the one which results in the highest modularity *Q*_*f*_ [15]:

(10) $$\begin{array}{c} Q_{f}=\frac{1}{2I}\underset{i,j}{\sum }\left[A_{ij}-\frac{k_{i}k_{j}}{2I}\right]\cdot s_{ij}, \end{array}$$

where *k*_*i*_ is the degree of node *i*, *I* is the total number of edges in the network, and *s*_*ij*_ = ∑_*k*=1_^*r*^ *U*_*ki*_ *U*_*kj*_.

## 3. Results and Discussion

### 3.1. Performance of NbHClust

**Figure 2:**
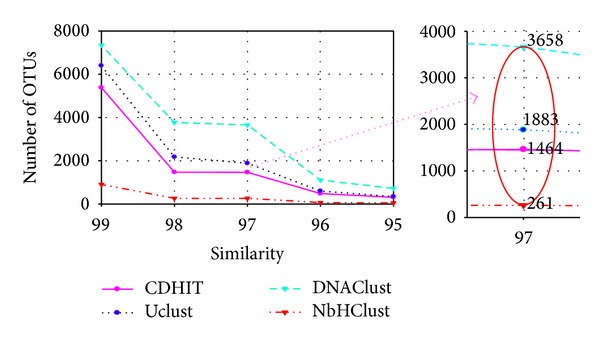
Results of four methods with Clone43 dataset.

In order to evaluate the performance of NbHClust, we compared NbHClust with the common used heuristic clustering methods CDHIT [16], Uclust [17], and DNAClust [18] on the Clone43 dataset [19], which consists of 202,340 reads from a mixture of 43 plasmid clones spanning the V6 region of 16S rRNA gene with an average length of 61 nt. Due to lack of ground truth, that is, species origin that each read belongs to is unknown, we used the number of OTUs estimated to evaluate the clustering quality.  Figure 2 shows the clustering results of four methods. From Figure 2, we can see that, at the commonly used threshold 97%, the smallest number of OTUs was ~260 returned by NbHClust, followed by Uclust (~1400), and CDHIT (~1900). The largest number was returned by DNAClust (~3700). These results show that NbHClust can reduce the OTU inflation and is much closer to the expected number (i.e., 43).

**Figure 3:**
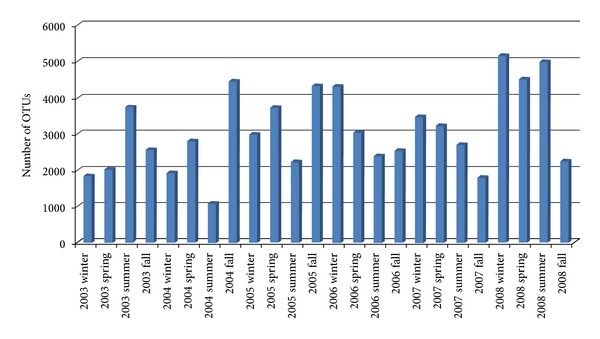
The distribution of seasonal microbial OTUs generated with NbHClust.

The number of seasonal microbial OTUs generated with NbHClust at 97% sequence identity is displayed in Figure 3, which shows that there are seasonal variations in OTU number throughout a 6-year period, and there are also repeating patterns.

### 3.2. Topology Analysis of Four Seasonal Marine Microbial Association Networks

**Figure 4:**
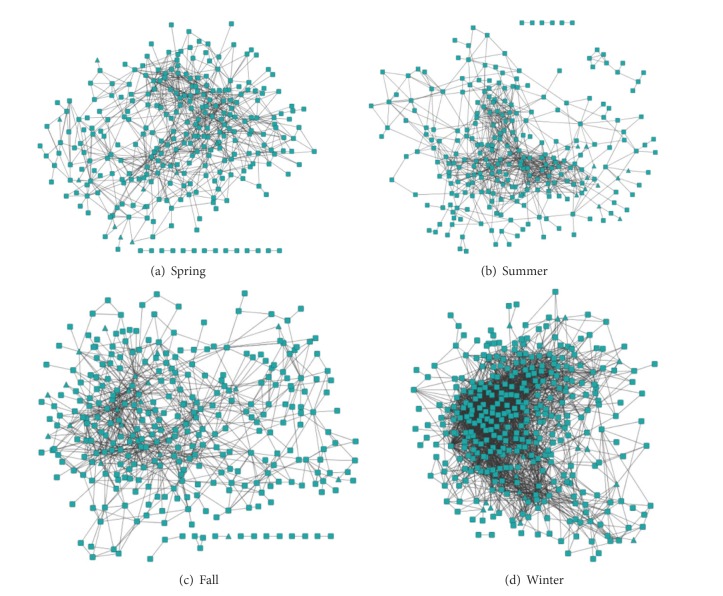
Marine microbial correlation networks in spring, summer, fall, and winter seasons (○-OTU, △-environmental factor).

In order to analyze the microbial diversity and the relationship among OTUs and environmental factors in spring, summer, fall, and winter seasons, we should construct the four seasonal marine microbial association networks. In general, mutual information (MI) provides a natural generalization of the correlation since it measures nonlinear dependency (which is common in biology) and has the ability to deal with thousands of variables (nodes). Although conditional mutual information (CMI) can detect the joint relationship of interesting variable (e.g., OTU) by two or more variables and other nonlinear interaction by two variables, its computational complexity is more than that of MI for large scale networks. Considering the number of OTUs and the computational time, we select MI to construct the four seasonal marine microbial networks. The four seasonal marine microbial association networks with MI algorithm are shown in Figure 4. We also computed their topological parameters including the average degree, average clustering coefficient, average power law degree, and modularity and compared them with their corresponding random networks. The comparison results of four seasonal networks and random networks are summarized in Table 1.

From Table 1, we can see that there is some difference in the topological parameters among the spring, summer, fall, and winter seasonal microbial correlation networks. Compared with random networks, four seasonal microbial correlation networks have bigger average clustering coefficient, average power law degree, and modularity, which indicate that the four seasonal microbial associate networks have some characters of complex network.

### 3.3. The Association Communities in Seasonal Microbial Networks Detected by s-NMF

**Figure 5:**
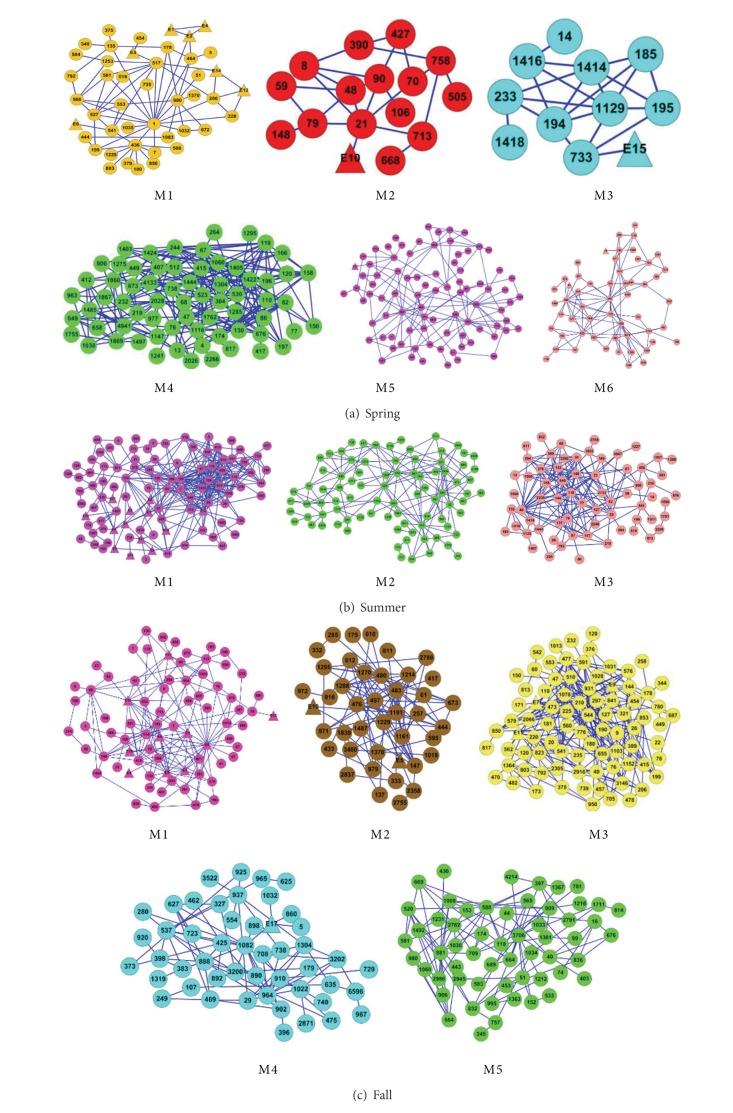

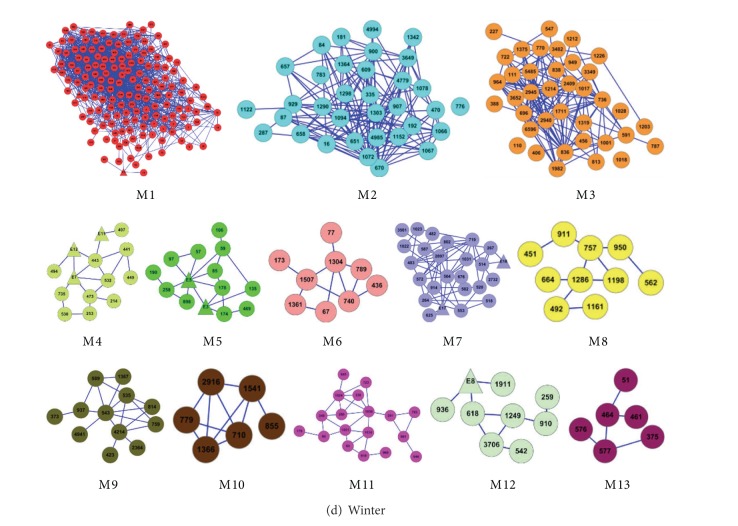
The structure of microbial interaction pattern detected by* s*-NMF algorithm in four seasonal networks. (○-OTU, △-environmental factor).

The four seasonal marine microbial association communities detected by* s*-NMF were shown in Figure 5. The results in Figure 5 show that the association community pattern diversity of winter is more than that of spring, summer, and fall, which indicates that the seasonal variability might have the greatest influence on the marine microbe diversity. We also find that some environmental factors are strongly associated with some microbes, and there are different association structures in four seasons. For instance, for M1 community in spring microbial network, the environmental factor E12 (NO_2_ + NO_3_) is correlative with OTU 206 (*Loktanella*) and OTU 228 (Alphaproteobacteria) and E14 (Silicate) are correlative with OTU 206 (*Loktanella*) and OTU 517 (Chloroplast). For M1 in summer microbial network, E12 (NO_2_ + NO_3_) is correlative with OTU 7 (*SAR*11), OTU 41 (*SAR*11), OTU 57 (*SAR*11), OTU 62 (*SAR*11), OTU 85 (*SAR*11), OTU 106 (*SAR*11), OTU 120 (*SAR*11), OTU 130 (*SAR*11), OTU 135 (*SAR*11), OTU 459 (*Haliea*), OTU 705 (*SAR*86), OTU 817 (Gammaproteobacteria), OTU 390 (Alphaproteobacteria), OTU 915 (*SAR*406), OTU 1036 (*Pseudospirillum*), and OTU 1980 (*Araneosa*); and E4 (DX2 = sin(2*π*(*d*/365))), where *d* is the number of days from December 20, is correlative with OTU 210 (Rhodobacteraceae), OTU 379 (*SAR*116), OTU 496 (*Fluviicola*), and OTU 1597 (*SAR*86); and E11 (Chlorophyll A) is correlative with OTU 3 (*SAR*11), OTU 9 (*SAR*11), OTU 418 (Rhodospirillaceae), and OTU 735 (*unknown*). For M1 in fall microbial network, E12 (NO_2_ + NO_3_) is correlative with OTU 14 (*SAR*11), OTU 92 (*SAR*11), OTU 130 (*SAR*11), OTU 406 (*SAR*116), OTU 342 (Rhodospirillaceae), OTU 459 (*Haliea*), OTU 1035 (Oceanospirillales), and OTU 789 (*Hellea*); and E16 (*temperature*) is correlative with OTU 1 (*Roseovarius*), OTU 68 (*SAR*11), OTU 82 (*SAR*11), OTU 92 (*SAR*11), OTU 158 (*SAR*11), OTU 294 (*SAR*86), OTU 534 (Chloroplast), OTU 418 (Rhodospirillaceae), OTU 456 (Alteromonadaceae), and OTU 789 (*Hellea*). For M4 in winter microbial network, E12 (NO_2_ + NO_3_) is correlative with OTU 494 (Cryomorphaceae) and OTU 443 (Chloroplast); and E7 (*primary production*) is correlative with OTU 443 (Chloroplast), OTU 473 (Chloroplast), OTU 532 (Chloroplast), and OTU 735 (*unknown*).

According to the annotation information of OTUs at taxonomic level by using a number of different annotation strategies (e.g., GAST [6], BLAST against Greengenes [20], SIVA [21], and RDP [22]), we analyzed in detail the OTU composition of community that included more environmental factors for every seasonal network.

The M1 community in spring microbial network is composed of 7 environmental factors (E1, E2, E4, E5, E6, E12, and E14) and 38 OTUs in which the 26 OTUs come from* Bacteria*, 11 come from organelle, and 1 OTU has not been annotated. In the 26* Bacteria* OTUs, 12 OTUs were identified in class level as Alphaproteobacteria, 6 OTUs as Gammaproteobacteria, 5 OTUs as* Flavobacteria*, and other three OTUs as Betaproteobacteria, Deferribacteres, Opitutae, respectively. In the 11 organelle OTUs, 10 OTUs come from Chloroplastand 1 OTU from Mitochondria.

The M1 community in summer microbial network is composed of 13 environmental factors (E1, E2, E3, E4, E5, E8, E9, E10, E11, E12, E14, E17, and E18) and 87 OTUs in which the 85 OTUs come from* Bacteria*, 1 come from Chloroplast, and 1 OTU has not been annotated. In the 85* Bacteria* OTUs, 47 OTUs were identified in class level as Alphaproteobacteria, 20 OTUs as Gammaproteobacteria, 6 OTUs as Flavobacteria, 3 OTUs as Deferribacteres, 2 OTUs as Betaproteobacteria, 2 OTUs as Verrucomicrobiae, and other OTUs as Actinobacteria, Clostridia, Cyanobacteria, Lentisphaeria, and Sphingobacteria, respectively.

The M1 community in fall microbial network is composed of 10 environmental factors (E1, E2, E3, E4, E6, E12, E14, E15, E16, and E18) and 65 OTUs in which the 59 OTUs come from* Bacteria* and 6 come from Chloroplast. In the 59* Bacteria* OTUs, 42 OTUs were identified in class level as Alphaproteobacteria, 9 OTUs as Gammaproteobacteria, 2 OTUs as Betaproteobacteria, 2 OTUs as Deltaproteobacteria, and other OTUs as Actinobacteria, Flavobacteria, Cyanobacteria,and Verrucomicrobiae, respectively.

The M1 community in winter microbial network is composed of 2 environmental factors (E4, E16) and 158 OTUs in which the 144 OTUs come from* Bacteria*, 12 come from Chloroplast, 1 comes from Crenarchaeota, and 1 comes from* unknown*. In the 144* Bacteria* OTUs, 95 OTUs were identified in class level as Alphaproteobacteria, 29 OTUs as Gammaproteobacteria, 4 OTUs as Betaproteobacteria, 7 OTUs as Deltaproteobacteria, 2 OTUs as Actinobacteria, 2 OTUs as* Bacilli*, 8 OTUs as Deferribacteres, 4 OTUs as Verrucomicrobiae, and other OTUs as Clostridia, Cyanobacteria,and Planctomycetacia, respectively.

The M4 community in winter microbial network is composed of 3 environmental factors (E7, E11, and E12) and 11 OTUs in which the 3 OTUs come from* Bacteria*, 7 come from* Chloroplast*, and 1 OTU has not been annotated. The 3* Bacteria* OTUs were identified in* family* level as Flavobacteria, Cryomorphaceae, and Rhodobacteraceae,respectively. The analysis results of other communities in the four season microbial networks can be found in the Supplementary Material (available online at http://dx.doi.org/10.1155/2014/189590).

The community structural analysis in four seasonal microbial networks shows that a large fraction microbial association in class level occurs among* Alphaproteobacteria* and* Gammaproteobacteria*; the community dense of summer, fall, and spring is bigger than that of winter; the correlative relationships are stronger between OTUs (taxa) than with environmental factors. This may indicate that biological rather than physical factors can be more important in defining the fine-grain community structure.

## 4. Conclusions

Mining the marine microbial association patterns and diversity is a key for exploiting the marine resources. Considering that the marine microbes are symbiosis or competition, exhibiting numerous, significant intra- or interlineage associations, we used the NbHClust and* s*-NMF approaches to analyze the potential association patterns between the marine microbes and environmental factors from the 16S rRNA sequences. The results show that the four seasonal marine microbial association networks have characters of complex networks, and the marine microbial association patterns are related to the seasonal variability; in the four seasons, the association between microbe and environmental factor is significantly different; that is, the same environmental factor influences the different species; and the correlative relationships are stronger between OTUs (taxa) than with environmental factors. Although we cannot claim that we have a comprehensive view of association within marine microbial communities, our analysis method is more feasible and interesting for exploring the unseen patterns that emerged in the complex dataset, including nonrandom association, deterministic processes at different taxonomic levels, and expected relationship between community members.

## Supplementary Material

The four seasonal marine microbial association communities are detected by s-NMF. The results show that the association community pattern diversity of winter is more than that of spring, summer and fall, which indicates that the seasonal variability might have the greatest influence on the marine microbe diversity. We also find that some environmental factors are strongly associated with some microbes, and there are different association structures in four seasons. Some typical communities are discussed in this paper, and the analysis results of other communities in four season microbial networks are shown with the Supplementary Material.

## Figures and Tables

**Table 1:** Topological parameters of four seasonal marine microbial correlational networks and the corresponding random networks.

	Seasonal networks				Random networks			
	Spring	Summer	Fall	Winter	1	2	3	4
Node number	280	254	313	365	280	254	313	365
Edge number	793	855	845	2970	793	855	845	2970
Avg. degree	5.664	6.732	5.399	16.274	5.664	6.732	5.399	16.274
Avg. clustering coefficient	0.235	0.282	0.237	0.389	0.010	0.026	0.022	0.046
Avg. power law degree	1.237	1.287	1.467	0.968	0.666	0.442	0.659	0.013
Modularity	0.579	0.567	0.561	0.365	0.39	0.34	0.404	0.217
