# Supplementary material for: Mining Seasonal Marine Microbial Pattern with Greedy Heuristic Clustering and Symmetrical Nonnegative Matrix Factorization
Source: Biomed Res Int. 2014 Apr 27;2014:189590. doi: 10.1155/2014/189590 (PMC4022257; doi:10.1155/2014/189590)
Supplement: Supplementary file 1 — The four seasonal marine microbial association communities are detected by s-NMF. The results show that the association community pattern diversity of winter is more than that of spring, summer and fall, which indicates that the seasonal variability might have the greatest influence on the marine microbe diversity. We also find that some environmental factors are strongly associated with some microbes, and there are different association structures in four seasons. Some typical communities are discussed in this paper, and the analysis results of other communities in four season microbial networks are shown with the Supplementary Material. [file 189590.f1.doc]

Appendix

Mining Seasonal Marine Microbial Pattern with

Greedy Heuristic Clustering and Symmetrical Non-negative Matrix Factorization

(ID:189590)

**Results of** **analyzing other communities in four season microbial networks**

**1. Spring marine microbial network**

The M1 community k is composed of 7 environmental factors (E1, E2, E4, E5, E6, E12, E14) and 38 OTUs in which the 26 OTUs come from *Bacteria*, 11 come from *Organelle* and 1 OTU has not been annotated. In the 26 Bacteria OTUs, 12 OTUs were identified in *Class* level as *Alphaproteobacteria*, 6 OTUs as *Gammaproteobacteria*, 5 OTUs as *Flavobacteria*, and other three OTUs as *Betaproteobacteria*, *Deferribacteres* , *Opitutae* respectively. In the 11 *Organelle* OTUs, 10 OTUs come from *Chloroplast* and 1 OTU comes from *Mitochondria*.

The M2 community is composed of 1 environmental factor (E10) and 15 OTUs that come from *Bacteria*. In the 15 Bacteria OTUs, 11 OTUs were identified in *Class* level as *Alphaproteobacteria*, 2 OTUs as *Gammaproteobacteria*, 1 OTU as *Flavobacteria*, and 1 OTU as *Betaproteobacteria*.

The M3 community is composed of 1 environmental factor (E15) and 10 OTUs that were identified in *Class* level as *Alphaproteobacteria*.

The M4 community is composed of 67 OTUs in which the 58 OTUs come from *Bacteria*, and 9 OTUS come from *Chloroplast*. In the 58 Bacteria OTUs, 40 OTUs were identified in *Class* level as *Alphaproteobacteria*, 8 OTUs as *Gammaproteobacteria*, 2 OTUs as *Flavobacteria*, 2 OTUs as *Bacilli*, 4 OTUs as *Sphingobacteria*, 1 OTUs as *Verrucomicrobiae*, and 1 OTUs as *Actinobacteria.*

The M5 community is composed of 1 environmental factor (E13) and 84 OTUs in which the 48 OTUs come from *Bacteria*, 31 OTUS come from *Chloroplast* 5 OTUs have not been annotated. In the 48 Bacteria OTUs, 12 OTUs were identified in *Class* level as *Alphaproteobacteria*, 13 OTUs as *Gammaproteobacteria*, 12 OTUs as *Flavobacteria*, 3 OTUs as *Actinobacteria*, 2 OTUs as *Cyanobacteria,* 3 OTUs as *Verrucomicrobiae,* and other two OTUs as *Betaproteobacteria*, *Sphingobacteria* respectively.

The M6 community is composed of 2 environmental factors (E3, E9) and 52 OTUs in which the 37 OTUs come from *Bacteria*, and 15 OTUS come from *Chloroplast*. In the 37 Bacteria OTUs, 8 OTUs were identified in *Class* level as *Alphaproteobacteria*, 17 OTUs as *Gammaproteobacteria*, 5 OTUs as *Flavobacteria*, 2 OTUs as *Actinobacteria*, 2 OTUs as *Deltaproteobacteria*, and other three OTUs as *Betaproteobacteria*, *Deferribacteres* , *Verrucomicrobiae* respectively.

**2. Summer** **marine microbial network**

The M1 community is composed of 13 environmental factors (E1, E2, E3, E4, E5, E8, E9, E10, E11, E12, E14, E17, E18) and 87 OTUs in which the 85 OTUs come from *Bacteria*, 1 comes from *Chloroplast* and 1 OTU has not been annotated. In the 85 *Bacteria* OTUs, 47 OTUs were identified in *Class* level as *Alphaproteobacteria*, 20 OTUs as *Gammaproteobacteria*, 6 OTUs as *Flavobacteria*, 3 OTUs as *Deferribacteres*, 2 OTUs as *Betaproteobacteria*, 2 OTUs as *Verrucomicrobiae*, and other OTUs as *Actinobacteria*, *Clostridia*, *Cyanobacteria*, *Lentisphaeria* and *Sphingobacteria* respectively.

The M2 community is composed 83 OTUs in which the 50 OTUs come from *Bacteria*, 32 come from *Chloroplast*, and 1 comes from unknown. In the 50 Bacteria OTUs, 9 OTUs were identified in *Class* level as *Alphaproteobacteria*, 7 OTUs as *Gammaproteobacteria*, 3 OTUs as *Deltaproteobacteria*, 4 OTUs as *Actinobacteria,* 17 OTUs as *Flavobacteria*,2 OTUs as *Sphingobacteria*, 3 OTUs as *Deferribacteres*, and 1 OTUs as *Planctomycetacia*.

The M3 community is composed of 65 OTUs in which the 59 OTUs come from *Bacteria*, 6 come from *Organelle.* In the 59 Bacteria OTUs, 39 OTUs were identified in *Class* level as *Alphaproteobacteria*, 11 OTUs as *Gammaproteobacteria*, 3 OTUs as *Flavobacteria,* 3 OTUs as *Verrucomicrobiae,* and other four OTUs as *Deltaproteobacteria, Betaproteobacteria*, *Sphingobacteria* and *Bacillales* respectively. In the 6 *Organelle* OTUs, 5 OTUs come from *Chloroplast* and 1 OTU from *Mitochondria*.

**3. Fall** **marine microbial network**

The M1 community is composed of 10 environmental factors (E1, E2, E3, E4, E6, E12, E14, E15, E16, E18) and 65 OTUs in which the 59 OTUs come from *Bacteria*, 6 come from *Chloroplast*. In the 59 Bacteria OTUs, 42 OTUs were identified in *Class* level as *Alphaproteobacteria*, 9 OTUs as *Gammaproteobacteria*, 2 OTUs as *Betaproteobacteria*, 2 OTUs as *Deltaproteobacteria*, and other OTUs as *Actinobacteria*, *Flavobacteria*, *Cyanobacteria* and *Verrucomicrobiae* respectively.

The M2 community is composed of 2 environmental factors (E5, E10) and 40 OTUs in which the 32 OTUs come from *Bacteria*, 6 come from *Chloroplas*, 2 come from unknown. In the 32 Bacteria OTUs, 10 OTUs were identified in *Class* level as *Alphaproteobacteria*, 5 OTUs as *Gammaproteobacteria*, 12 OTUs as *Flavobacteria*, 2 OTUs as *Deltaproteobacteria*, and other three OTUs as *Actinobacteria*, *Sphingobacteria* , and *Verrucomicrobiae* respectively.

The M3 community is composed of 3 environmental factors (E7, E9, E11) and 81 OTUs in which the 68 OTUs come from *Bacteria*, 13 come from *Chloroplast*. In the 68 Bacteria OTUs, 37 OTUs were identified in *Class* level as *Alphaproteobacteria*, 12 OTUs as *Gammaproteobacteria*, 2 OTUs as *Betaproteobacteria*, 4 OTUs as *Deltaproteobacteria*, 5 OTUs as *Flavobacteria*, 4 OTUs as *Verrucomicrobiae,* 2 OTUs as *Cyanobacteria*, and other two OTUs as *Actinobacteria*, and *Clostridiales* espectively.

The M4 community is composed of 1 environmental factor (E17) and 44 OTUs in which the 41 OTUs come from *Bacteria*, 3 come from *Chloroplast*. In the 41 Bacteria OTUs, 12 OTUs were identified in *Class* level as *Alphaproteobacteria*, 9 OTUs as *Gammaproteobacteria*, 6 OTUs as *Deltaproteobacteria*, 2 OTUs as *Actinobacteria*, 6 OTUs as Deferribacteres, 3 OTUs as *Verrucomicrobiae*, and other three OTUs as *Betaproteobacteria*, *Flavobacteria*, and *Cyanobacteria* respectively.

The M5 community is composed of 49 OTUs in which the 47 OTUs come from *Bacteria*, 1 comes from *Organelle* and 1 comes from *Archaea*. In the 47 Bacteria OTUs, 15 OTUs were identified in *Class* level as *Alphaproteobacteria*, 18 OTUs as *Gammaproteobacteria*, 3 OTUs as *Deltaproteobacteria*, 5 OTUs as *Flavobacteria*, 3 OTUs as *Verrucomicrobiae* and other three OTUs as *Betaproteobacteri*, *Actinobacteria* and Sphingobacteria respectively.

**4. Winter marine microbial network**

The M1 community is composed of 2 environmental factors (E4, E16) and 158 OTUs in which the 144 OTUs come from *Bacteria*, 12 come from *Chloroplast*, 1 comes from *Crenarchaeota*, 1 comes from unknown. In the 144 Bacteria OTUs, 95 OTUs were identified in *Class* level as *Alphaproteobacteria*, 29 OTUs as *Gammaproteobacteria*, 4 OTUs as *Betaproteobacteria*, 7 OTUs as *Deltaproteobacteria*, 2 OTUs as *Actinobacteria,* 2 OTUs as *Bacilli*, 8 OTUs as *Deferribacteres*, 4 OTUs as *Verrucomicrobiae*, and other OTUs as *Clostridia*, *Cyanobacteria* and *Planctomycetacia* respectively.

The M2 community is composed of 34 OTUs in which the 32 OTUs come from *Bacteria*, and 2 come from *Chloroplast*. In the 32 Bacteria OTUs, 8 OTUs were identified in *Class* level as *Alphaproteobacteria*, 17 OTUs as *Gammaproteobacteria*, 2 OTUs as *Bacilli*, and other five OTUs as *Betaproteobacteria*, *Deltaproteobacteria*, *Flavobacteria*, *Verrucomicrobiae*, and *Clostridiales* respectively.

The M3 community k is composed of 36 OTUs in which the 32 OTUs come from *Bacteria*, 1 comes from *Chloroplast*, and 3 come from unknown. In the 32 Bacteria OTUs, 6 OTUs were identified in *Class* level as *Alphaproteobacteria*, 12 OTUs as *Gammaproteobacteria*, 2 OTUs as *Deltaproteobacteria*, 4 OTUs as *Actinobacteria,* 3 OTUs as *Verrucomicrobiae*, 2 OTUs as *Planctomycetacia*, and other four OTUs as *Flavobacteria*, *Deferribacteres*, *Clostridia*, and *Gemmatimonadetes* respectively.

The M4 community is composed of 3 environmental factors (E7, E11, E12) and 11 OTUs in which the 3 OTUs come from *Bacteria,* 7 come from *Chloroplast* and 1 OTU has not been annotated. The 3 *Bacteria* OTUs were identified in *Family* level as *Flavobacteria*, *Cryomorphaceae* and *Rhodobacteraceae* respectively.

The M5 community is composed of 2 environmental factors (E3, E5) and 12 OTUs in which they are both come from *Bacteria*. In the 12 Bacteria OTUs, 9 OTUs were identified in *Class* level as *Alphaproteobacteria*, 2 OTUs as *Flavobacteria*, and 1 OTU as *Gammaproteobacterias*.

In the M6 community is composed of 9 OTUs which are *Bacteria*. In the 9 Bacteria OTUs, 6 OTUs were identified in *Class* level as *Alphaproteobacteria*, 2 OTUs as *Gammaproteobacteria*, and 1 OTU as *Flavobacteria*.

The M7 community is composed of 2 environmental factors (E17, E18) and 23 OTUs in which the 12 OTUs come from *Bacteria*, and 11 come from *Chloroplast*. In the 12 Bacteria OTUs, 2 OTUs were identified in *Class* level as *Alphaproteobacteria*, 3 OTUs as *Gammaproteobacteria*, 4 OTUs as *Flavobacteria*, 3 OTUs as *Actinobacteria* and 2 OTUs as *Deferribacteres*.

The M8 community is composed of 10 OTUs in which the 7 OTUs come from *Bacteria*, and 3 come from *Chloroplast*. In the 7 Bacteria OTUs, 2 OTUs were identified in *Class* level as *Alphaproteobacteria*, 2 OTUs as *Gammaproteobacteria*, 2 OTUs as *Flavobacteria*, and 1 OTU as *Verrucomicrobiae*.

The M9 community is composed of 11 OTUs in which the 7 OTUs come from *Bacteria*, 3 come from *Chloroplast*, and 1 comes from unknown. In the 7 Bacteria OTUs, 4 OTUs were identified in *Class* level as *Gammaproteobacteria*, 2 OTUs as *Actinobacteria,* 1 OTU as *Gemmatimonadetes*.

The M10 community is composed of 6 OTUs in which the 4 OTUs come from *Bacteria*, 2 come from *Chloroplast*. In the 6 Bacteria OTUs, they are both identified in *Class* level as *Gammaproteobacteria*.

The M11 community is composed of 18 OTUs which are *Bacteria*. In the 18 Bacteria OTUs, 3 OTUs were identified in *Class* level as *Alphaproteobacteria*, 6 OTUs as *Gammaproteobacteria*, 3 OTUs as *Deltaproteobacteria*, 3 OTUs as *Deferribacteres*, 2 OTUs as *Actinobacteria*, and 1 OTU as *Bacilli*.

The M12 community is composed of 1 environmental factor (E8) and 8 OTUs in which the 5 OTUs come from *Bacteria*, and 3 come from *Chloroplast*. In the 5 Bacteria OTUs, 2 OTUs were identified in *Class* level as *Gammaproteobacteria*, 2 OTUs as *Deltaproteobacteria*, and 1 OTU as *Flavobacteria*.

The M13 community is composed of 6 OTUs which are *Bacteria*. In the 6 Bacteria OTUs, 2 OTUs were identified in *Class* level as *Alphaproteobacteria*, 2 OTUs as *Gammaproteobacteria*, and 2 OTUs as *Cyanobacteria*.
